# Burden of paediatric respiratory syncytial virus disease and potential effect of different immunisation strategies: a modelling and cost-effectiveness analysis for England

**DOI:** 10.1016/S2468-2667(17)30103-2

**Published:** 2017-07-31

**Authors:** Deborah Cromer, Albert Jan van Hoek, Anthony T Newall, Andrew J Pollard, Mark Jit

**Affiliations:** a Kirby Institute for Infection and Immunity in Society, University of New South Wales, Sydney, NSW, Australia; b Department of Infectious Disease Epidemiology, London School of Hygiene & Tropical Medicine, London, UK; c School of Public Health and Community Medicine, University of New South Wales, Sydney, NSW, Australia; d Oxford Vaccine Group, Department of Paediatrics, University of Oxford, Oxford, UK; e National Institute of Health Research, Oxford Biomedical Research Centre, Children's Hospital, Oxford, UK; f Modelling and Economics Unit, Public Health England, London, UK

## Abstract

**Background:**

Vaccines and prophylactic antibodies against respiratory syncytial virus (RSV) are in development and likely to be available in the next 5–10 years. The most efficient way to use these products when they become available is an important consideration for public health decision makers.

**Methods:**

We performed a multivariate regression analysis to estimate the burden of RSV in children younger than 5 years in England (UK), a representative high-income temperate country, and used these results to assess the potential effect of different RSV immunisation strategies (targeting vaccination for infants, or pregnant women, or prophylactic antibodies for neonates). We did a cost-effectiveness analysis for these strategies, implemented either separately or concurrently, and assessed the effect of restricting vaccination to certain months of the year.

**Findings:**

We estimated that RSV is responsible for 12 primary care consultations (95% CI 11·9–12·1) and 0·9 admissions to hospital annually per 100 children younger than 5 years (95% CI 0·89–0·90), with the major burden occurring in infants younger than 6 months. The most cost-effective strategy was to selectively immunise all children born before the start of the RSV season (maximum price of £220 [95% uncertainty interval (UI) 208–232] per vaccine, for an incremental cost-effectiveness ratio of £20 000 per quality-adjusted life-year). The maximum price per fully protected person that should be paid for the infant, newborn, and maternal strategies without seasonal restrictions was £192 (95% UI 168–219), £81 (76–86), and £54 (51–57), respectively.

**Interpretation:**

Nearly double the number of primary care consultations, and nearly five times the number of admissions to hospital occurred with RSV compared with influenza. RSV vaccine and antibody strategies are likely to be cost-effective if they can be priced below around £200 per fully protected person. A seasonal vaccination strategy is likely to provide the most direct benefits. Herd effects might render a year-round infant vaccination strategy more appealing, although it is currently unclear whether such a programme would induce herd effects.

**Funding:**

UK National Institute for Health Research.

## Introduction

Respiratory syncytial virus (RSV) is a highly seasonal respiratory virus (the season runs from from late autumn to early spring).^1^ Exposure to RSV does not lead to long-lasting protection and hence people can have many infections over their lifetime.^2^ Infection mainly leads to mild disease, but in very young children (aged <6 months), elderly people, and immunocompromised patients it can result in serious disease or death.^3^

Currently, the only effective preventive strategy against RSV is passive immunisation with palivizumab, a humanised monoclonal RSV-specific antibody. Because of its high price, this antibody is only used in the highest-risk groups of individuals during the RSV season (November to February)—usually young children who are born prematurely and have other respiratory or cardiac conditions.^4^, ^5^ However, around 60 RSV vaccine and monoclonal antibody candidates are in development, 16 of which are in clinical trials,^6^, ^7^ although trial results in adults aged 60 years and older for the most advanced vaccine candidate (Resolve RSV-F vaccine) have not shown efficacy.^8^ Besides older adults, other potential candidates are pregnant women (to protect newborn babies through passive immunity), newborn babies (through passive immunisation with antibodies), and infants. An RSV vaccine could possibly be licensed in the next 5–10 years.^9^ Additionally, at least one extended, half-life monoclonal antibody designed to protect infants from birth, along with at least three maternal vaccines, are in clinical trials.^6^

Decision makers will need to understand the potential health and economic effects of the different vaccine and antibody options to select strategies that maximise the effect of health-care resources. Although the exact characteristics of future maternal or infant vaccines or prophylactic antibodies for newborn babies are unknown, understanding the burden of RSV disease and the drivers of vaccine effects and value can help to inform decisions about prioritisation of vaccination or antibody strategies, and protocols for clinical trials.^7^ Such analyses can also identify and help to ensure that data are obtained in advance about the key drivers of cost-effectiveness.

**Research in context**

**Evidence before this study**

Respiratory syncytial virus (RSV) disease is the primary contributor to childhood lower respiratory tract infections. More than 60 biological candidates for RSV prophylaxis (vaccines and prophylactic monoclonal and polyclonal antibodies) are undergoing development, of which more than 25% have progressed to human trials, and one or more is likely to be licenced in the next 5–10 years. The candidates target different patient populations, and the optimum prophylactic strategy is yet to be determined. We did a search of the scientific literature, based on expert opinions. Despite some previous studies separately assessing the incidence of RSV-attributable clinical disease, and the economic impact of vaccination, as yet there have been no studies that combine this information, and few published studies can be used by decision-making bodies to assess the cost-effectiveness of different RSV vaccination strategies.

**Added value of this study**

We used data from laboratory reports and on health-care attendances for acute respiratory illness to estimate disease burden and health-care costs associated with RSV in England (UK). The estimates agreed with those from previous studies, while providing greater insight into the timing of outbreaks and ages most affected. We present the first quantitative analysis to highlight how the month of birth affects RSV-attributable health-care outcomes in a temperate climate. We then assessed the effect and cost-effectiveness of various vaccine and antibody strategies in pregnant women and young children. We showed that children born immediately before the RSV season, which runs from late autumn to early spring, have a two-fold higher risk of primary-care attendance and a four-fold higher risk of being admitted to hospital than children born after the season.

**Implications of all the available evidence**

Given the difference in these risks between children born before and after RSV season, the most cost-effective strategies, and ones that have the potential to avert the most severe disease and deaths, are those that protect children born just before the RSV season, such as maternal vaccination or long-lasting prophylactic monoclonal antibodies.

So far, few studies are available to inform about the potential cost-effectiveness of different RSV vaccination strategies^10^ and the need for further cost-effectiveness information has been identified as a priority by WHO's Strategic Advisory Group of Experts on Immunisation.^11^ To help to address this need, we present a detailed analysis of the disease burden of RSV and the associated health-care costs in England (UK). We then used England as an example of a high-income country in the temperate zone that is considering RSV vaccination in the future. This allowed us to illustrate general principles and to explore the potential effect and cost-effectiveness of different vaccine and antibody strategies to protect young children in high income, temperate climates with a similar epidemiology to England.

## Methods

### Disease burden estimation

Most people who present to health-care services with respiratory symptoms are not routinely tested for RSV, so the incidence of primary care attendances and hospital admissions for RSV has to be inferred. We used a statistical regression model^12^ to ecologically link clinical attendances for acute respiratory infection to organisms detected in routine clinical microbiological testing, based on temporal trends in both datasets, similar to our work estimating the burden of seasonal influenza.^12^ Similar methods have been previously used to explore the burden of seasonal organisms, including RSV, influenza virus, and rotavirus.^13^, ^14^ We synthesised information from general practice attendances and hospital admissions for acute respiratory symptoms and positive laboratory reports for respiratory pathogens with data from the scientific literature^15^ to explore the detailed age distribution of these clinical attendances in children younger than 5 years. Full details of our methods are in the appendix (p 1).

### Economic model

We used a static cohort model (ie, a model that does not account for the indirect or herd effects of vaccination) to explore the potential direct effect of paediatric vaccination or long-lasting monoclonal antibody use on its recipient (appendix p 5). We used the results of the model to estimate the net cost and cost-effectiveness of the interventions. We estimated the maximum cost-effective price (MCEP) per fully protected individual that could be paid for both the purchase and the administration costs of a course of vaccines or prophylactic antibodies (including any required booster doses), so as not to exceed the threshold of £20 000 per quality-adjusted life-year (QALY) gained, which is commonly used as a measure of cost-effectiveness in England.^16^ This value is close to the UK's gross domestic product per capita, which has been suggested^17^ as a possible threshold to use for an intervention to be deemed very cost-effective. The maximum price payable for each fully vaccinated individual for a range of assumptions on vaccine efficacy is in the appendix (p 10). Further details including cost-related and health-related quality-of-life parameters are in table 1, and the appendix (p 9).

**Table 1:** Base case parameters for the cost-effectiveness model and variations used in the sensitivity analysis

	**Base case value**	**Lower limit**	**Upper limit**
Vaccine efficacy*	70%	50%	100%
Age of infant vaccine administration	3 months	2 months	4 months
Duration of maternal antibody protection	3 months	2 months	4 months
Duration of neonatal prophylactic antibody protection	6 months	4 months	8 months
Multiplier for deaths	1·0	0	2·0
Multiplier for QALYs	1·0	0·8	1·2
Multiplier for costs	1·0	0·8	1·2
Discounting	3·5%	1·5%	3·5%

QALY=quality-adjusted life-year.

* Because there were no available data to inform on vaccine efficacy, we chose a mid-range efficacy value.

### Interventions

We considered vaccination strategies that targeted either infants, pregnant women, or neonates. We assumed that neonates would be protected either through passive immunisation via maternal vaccination, anticipated to give 3 months' protection, or through an extended half-life monoclonal antibody administered to newborn infants and providing passive immunisation, anticipated to give 6 months' protection. We also considered the strategy of vaccinating neonates born in certain months of the year only (appendix p 7). Assumptions behind all vaccination strategies are in the appendix (p 7).

### Statistical analysis

We ran a probabilistic sensitivity analysis, varying the number of RSV-attributable cases, costs, and QALYs (table 2, appendix). 95% uncertainty intervals (UIs) are the result of 10 000 simulations. Additionally, to determine the sensitivity of our cost-effectiveness estimates to different model variables, we ran a sensitivity analysis, sequentially altering model parameters from the baseline values. We did all analyses with R version 3.2.2.

**Table 2:** Estimated number of annual general practitioner (GP) consultations, admissions to hospital, and deaths in hospital attributable to respiratory syncytial virus in children younger than 5 years

		**Age less than 6 months**	**Age 6 months to less than 5 years**	**Age less than 5 years**
GP consultations		64 570 (63 700–65 430)	288 000 (284 230–291 770)	352 570 (348 700–356 440)
	Incidence per 100 population	21·42 (21·13–21·70)	10·92 (10·77–11·06)	11·99 (11·86–12·12)
Admissions to hospital		13 250 (13 200–13 310)	13 150 (13 020–13 280)	26 400 (26 270–26 550)
	Incidence per 100 population	4·4 (4·38–4·41)	0·5 (0·49–0·50)	0·9 (0·89–0·90)
GP consultations leading to hospital admissions		20·53%	4·57%	13·00%
Deaths in hospital		7·49 (7·19–7·79)	17·39 (16·92–17·86)	24·88 (24·32–25·44)
	Incidence per 100 population	0·00248 (0·00238–0·00258)	0·00066 (0·00064–0·00068)	0·00085 (0·00083–0·00087)

Data are n (95% CI), incidence per 100 population (95% CI), or n (%). 95% CIs were used to fit a normal distribution in the probabilistic sensitivity analysis.

### Role of the funding source

The funder of the study had no role in study design, data collection, data analysis, data interpretation, or writing of the report. The corresponding author (DC) had full access to all the data in the study and had final responsibility for the decision to submit for publication.

## Results

**Figure 1:**
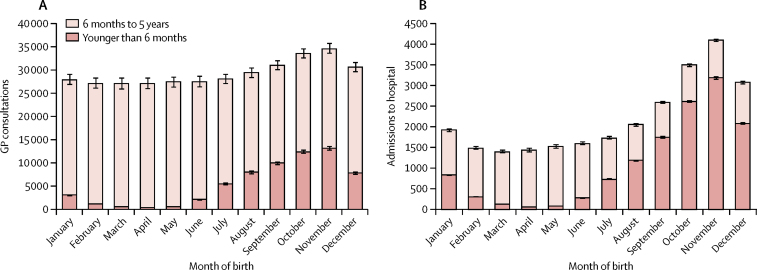
Outcomes attributable to respiratory syncytial virus by month of birth (A) General practitioner (GP) consultations, (B) admissions to hospital.

Variations in baseline parameters are in table 1. Results from the regression model suggested that 352 570 (16%) of 2 217 400 acute respiratory general practitioner (GP) consultations and 26 400 (22%) of 122 100 admissions to hospital for acute respiratory conditions are attributable to RSV in children younger than 5 years (table 2). We estimated that RSV is responsible for around 12 primary care consultations (95% CI 11·86–12·12) and 0·9 admissions to hospital annually per 100 children younger than 5 years (95% CI 0·89–0·90), with the major burden occurring in infants younger than 6 months (table 2, figure 1). In children younger than 6 months, RSV accounted for more than half of all admissions to hospital for acute respiratory conditions and for more than 70% of those admissions occurring between October and January (table 2). We estimated that there were around 25 deaths (95% CI 24·32–25·44) in children younger than 5 years (table 2). Although proportionally, the number of RSV-attributable outcomes was higher in children younger than 6 months, in absolute terms most of the burden occurred in children aged 6 months to 5 years (table 2).

RSV is extremely seasonal, with peaks of incidence in December and January that predominantly affected children younger than 6 months (appendix p 3). Children born in winter had more RSV-attributable GP consultations and admissions to hospital (figure 1), and a higher proportion of their primary care outcomes occurred when they were younger than 6 months (dark red shading in figure 1), similar to that previously reported for RSV-attributable laboratory reports.^1^ The incidence of RSV-attributable GP consultations in the first year of life fluctuated between 13·9 per 100 children born in March up to 27·4 per 100 children born in November (and similarly from 1·55 per 100 births to 6·47 per 100 births for admissions to hospital).

Health-care costs for RSV in children younger than 5 years are £54 million annually (95% UI 50 million–57 million) or £87·58 annually per child (82–93). Most of this cost (£37 million) resulted from RSV-attributable admissions to hospital (including admissions to intensive care, which are assumed to be more likely in preterm infants), and was split approximately equally between children younger than 6 months (£19·1 million [95% UI 18·7 million–19·4 million]) and children aged between 6 months and 5 years (£18·4 million [16·3 million–20·4 million]). RSV-attributable GP consultations cost the health-care service £16 million annually (95% UI 14 million–19 million), with £13 million (82%) of the total cost (£16 million) for older children and £3 million (18%) for children younger than 6 months. Almost 70% (3526 of 5221) of QALYs lost stemmed from events associated with GP consultations and almost 75% (3841 of 5221) of QALY losses were caused by outcomes in older children.

**Figure 2:**
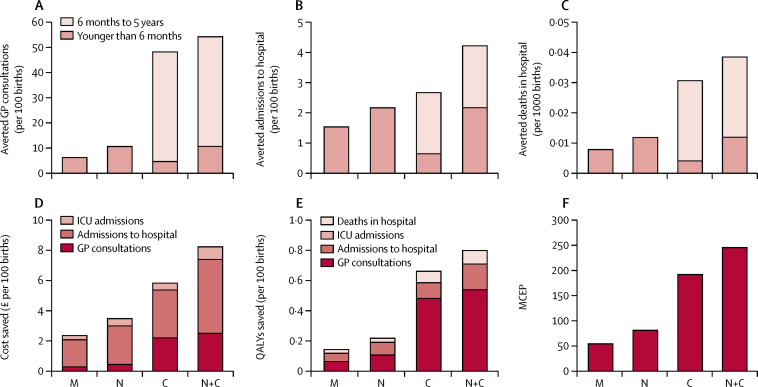
Cases of respiratory syncytial virus averted and costs or QALYs saved for different vaccination strategies with complete vaccine efficacy Data given per 100 annual births for (A) general practitioner (GP) consultations averted,(B) hospital admissions averted, (C) deaths in hospital averted, (D) health-care costs saved, (E) QALYs saved, and (F) maximum cost-effective price (MCEP) of vaccination strategy. M=maternal immunisation strategy. N=newborn passive immunisation strategy. C=infant strategy, N+C=newborn and infant strategies. ICU=intensive-care unit. QALY= quality-adjusted life-year.

Results of vaccination are shown for a vaccine with 100% efficacy to present the maximum possible effect; however, the proportional effect from different strategies was the same regardless of the vaccine efficacy (figure 2). Most of the cost-savings from any strategy resulted from averted admissions to hospital and intensive care (62% using the infant strategy and 86–88% when protecting newborn babies; figure 2), despite most of the averted cases being in primary care (95%, 83%, and 80% for infant, newborn, and maternal strategies, respectively). QALY gains from all strategies were driven largely by averting GP consultations (73% of gains for an infant strategy and 47% and 43% for newborn and maternal strategies, respectively; figure 2). For all strategies, just over 10% of the QALYs gained were from avoidance of RSV-attributable deaths in hospital. With the same vaccine efficacy, a newborn strategy averted many more RSV-attributable outcomes in babies younger than 6 months than a programme for infants aged 3 months and older.

In the base case, the maximum price payable per fully protected person that should be paid for infant, newborn, and maternal vaccination strategies without seasonal restrictions was £192 (95% UI 168–219), £81 (76–86), and £54 (51–57), respectively.

The MCEP for a strategy that combined a newborn and infant programme was £246 (95% UI 219–275). However, if a newborn programme was already in place (hence reducing disease burden and thus the benefit of any further immunisation strategies), then the MCEP of an infant vaccine would drop from £192 to £165 (143–190). Likewise, if an infant programme was already in place, then the MCEP for a newborn strategy decreased from £81 to £54 (51–57).

**Figure 3:**
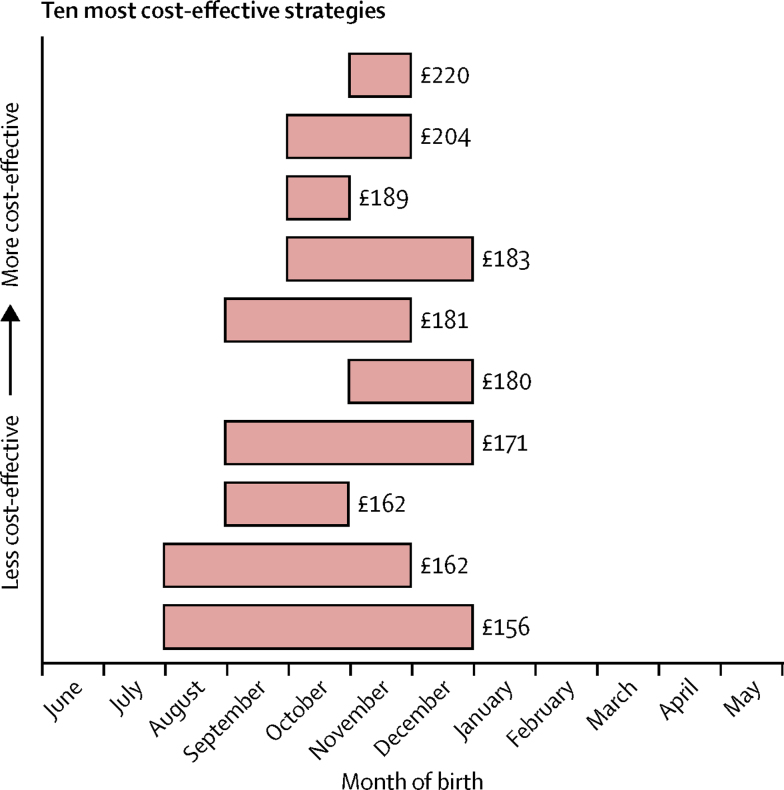
Ten most cost-effective periods over which to offer newborn vaccination or prophylactic antibodies against respiratory syncytial virus Red shading shows the birth months of the children that are most cost-effective to protect via a newborn strategy. Prices are per fully protected child for vaccination or prophylactic antibodies. All of the ten most cost-effective programmes involved protecting newborn infants for 5 months or less of the year, the top nine protected for 4 months or less, and the top eight recommended protecting children born in November.

Regardless of the actual cost of an immunisation strategy or its efficacy, because of the extreme seasonality of RSV, and its propensity to infect very young children, a strategy to protect newborn babies is most cost-effective if it is only administered during certain months of the year. In the UK, the most cost-effective strategy was to protect only neonates born in November (before the start of the RSV season; MCEP of £220 [95% UI 208–232] per fully protected newborn infant). We noted that nine of the top ten most cost-effective strategies involved restricting prophylaxis to neonates born in only 4 months (or fewer) of the year, and who were born before the peak in RSV incidence (figure 3).

**Figure 4:**
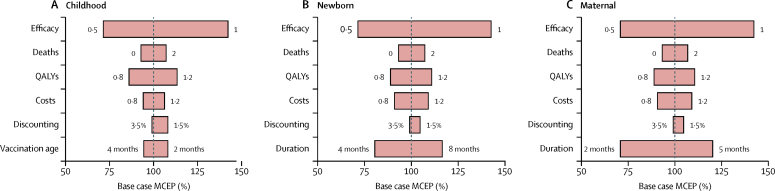
Sensitivity to model parameters of cost-effectiveness calculations for respiratory syncytial virus vaccination (A) Infant strategy. (B) Newborn infant strategy. (C) Maternal strategy. Bars show by how much the maximum cost-effective price changes from its base case level when model parameters are varied. Changing the discounting strategy, or excluding deaths from the model, had little effect on the maximum cost-effective price (MCEP) estimates. Similarly, modifying the costs or quality-adjusted life-years (QALYs) associated with each health-care outcome had little effect on model estimates.

The model was most sensitive to vaccine efficacy, and for the maternal or newborn strategy, to the duration of vaccine protection (figure 4). It was difficult to predict at this stage the potential effect that an RSV vaccine or antibody might have on infection transmission and hence indirect (herd) benefits, so the results presented here provide a conservative lower bound of the maximum price to pay per protected person, in the absence of consideration of herd effects. To estimate the maximum benefit that might be conferred through indirect effects, we made the assumption that introduction of a vaccine would completely eliminate disease transmission and hence disease in all children younger than 5 years (including those too young to receive the vaccine). Under this assumption, the maximum price for a full course and administration of a vaccine was £246 (220–276).

## Discussion

There is a large and costly RSV disease burden in children younger than 5 years, especially infants younger than 6 months (particularly for admissions to hospital) and in the winter months. Indeed, in children younger than 5 years RSV is responsible for nearly twice as many GP consultations and nearly five times as many admissions to hospital as influenza, for which paediatric vaccination has been found to be cost-effective.^18^ RSV accounts for more than 75% of infants admitted to hospitals for respiratory conditions between the beginning of November and the end of January, consistent with other studies showing RSV to be a leading cause of infant admissions to hospital.^19^ We have shown how RSV-attributable health-care outcomes vary based on month of birth, with children born just before the start of the RSV season having double the risk of an RSV-attributable GP consultation and more than a four-fold higher risk of an RSV-attributable admission to hospital in their first year of life than those born after the RSV season. The general trends and conclusions for England are likely to be similar in high-income temperate countries with similar epidemiology, widespread health services and existing, well supported vaccination programmes.

Our estimates for RSV-attributable admissions to hospital are in line with other UK estimates,^13^, ^20^, ^21^ and many reported in western Europe,^22^, ^23^ but lower than estimates reported for Spain (a full comparison is in the appendix p 8). Our findings about the marked seasonality of RSV disease agree with those from a study in England^1^ showing that infants born just before or during the RSV season had a much higher risk of laboratory-confirmed RSV in their first year than those born just after the RSV season.^1^

Around 20% of infants visited GPs for RSV-attributable consultations in the first 6 months of life, and a fifth of these were admitted to hospital. Although the burden of RSV decreased as infants matured, nearly half of all children aged 6 months to 5 years visited GPs for RSV-attributable respiratory illnesses, with 5% resulting in admissions to hospital. These estimates agree with those from a study^13^ based on a restricted regression analysis of UK data that only incorporated laboratory reports for influenza and RSV rather than all respiratory pathogens.

One of the limitations of our study was that our disease estimates were based on statistical models, similar to those used to understand the burden of other respiratory^12^, ^13^ and diarrhoeal diseases.^14^ Therefore the normal caveats apply, such as difference in sensitivity between tests, reporting bias, testing practices, and unattributable changes over time. However, our RSV model was based on previous work testing nine models incorporating adjustments suggested by others^24^, ^25^ on six different age groups and selected the best-fitting model.^12^ Our assumptions of the effects that RSV infection has on quality of life, although from a previous cost-effectiveness analysis,^10^ were based on expert opinion rather than data. Indeed, RSV-averted deaths through vaccination might have occurred in children who had a lower quality of life or shorter life expectancy than average because of other comorbidities, and this differential might make vaccination less cost-effective. Future cost-effectiveness studies could benefit from better understanding of the effect of RSV disease on quality of life in young children, more detailed information on RSV incidence by month of birth in children younger than 1 year, and more detailed information about disease in preterm infants. Our burden estimates were based on data from 2001 to 2008, and although they were in agreement with other estimates, all were done before the introduction of paediatric vaccination for influenza; thus, future studies are required to consider the implications of this policy change on RSV burden.

The exact effect of an RSV vaccine or monoclonal antibody depends heavily on the age at which it can be given, and the age profile of RSV disease burden in very young infants. We based our analysis on several existing studies which showed RSV-associated admissions to hospital peaked at around 2 months of age, and decreased thereafter.^15^, ^26^, ^27^ However, RSV probably induces more severe disease in younger age groups^28^ and therefore the age profile of milder disease might be different. Understanding both the age-distribution and seasonality of RSV disease is key to selecting the best preventive strategy; hence, further direct active surveillance is needed to get better estimates. Additionally, although the burden of RSV in low-income and middle-income countries is substantial,^22^ further work is needed to assess the effect of interventions in these settings because of differences in seasonality of disease, access to care, resources available to pay for interventions, and population comorbidities.

Since neither the mechanism of action or the efficacy of RSV-immunisation strategies for either newborn babies or infants are known, a transmission model was not used in this analysis, therefore herd effects that might protect infants too young to be vaccinated, other unvaccinated children, and older individuals could not be assessed. If an RSV vaccine can prevent transmission as well as disease, the vaccine is likely to be even more cost-effective than our analysis suggests, and the results of future clinical trials will be essential to determine vaccine efficacy for each strategy. Using an assumption of complete disease elimination in children younger than 5 years, we concluded that the maximum price payable for the full purchase and administration of an RSV-immunisation programme would be £244. Herd effects might thus render a year-round vaccination strategy more cost-effective than a seasonal one, since a seasonal strategy is unlikely to elicit these effects. Hence, once suitable data on vaccine mechanisms become available the cost-effectiveness should be reassessed using a dynamic model. Additionally, because of the uncertainties described above, and the uncertainty in vaccine price, we did not use the traditional approach of comparing the cost-effectiveness of different options incrementally to each other, since this would require knowing the cost of each option. Once further details of the vaccines become available, a full incremental cost-effectiveness analysis of all options together would be helpful.

We did not consider the vaccination of children older than early infancy. Such a strategy would not directly protect infants at the age of highest disease incidence, but might have a large effect on disease by protecting the younger group through herd (indirect) protection.

The most cost-effective strategy assessed was a seasonal strategy that protected children who are born just before the RSV season from birth for the first few months of life. The exact month of vaccination should be determined on the basis of the epidemiology of each country. Such a seasonal vaccination strategy would probably only be feasible for a single-dose immunisation strategy, either given to the mother before birth, or to a child in the first few weeks of life. This suggests that efforts focused on developing an efficacious maternal vaccine, or a birth dose of a long-lasting monoclonal antibody, and on investigating the potential for vertical protection are well placed. Single-dose prophylactic antibodies have completed phase 1 trials in adults^29^ and are in phase 1b and 2a clinical trials in infants.^6^

Under certain conditions, protecting older infants would be more cost-effective than protecting neonates. However, these conditions only hold under optimistic assumptions about an infant vaccine—ie, that it will confer full protection from age 6 months to 5 years compared with the rapidly waning protection from a newborn dose of monoclonal antibody or maternal immunisation. Additionally, we showed that most QALY gains from vaccination were attributable to avoiding GP consultations rather than hospital admissions and deaths. This result drives the greater relative economic value of infant strategies compared with newborn or maternal strategies, even though maternal strategies might prevent more severe RSV cases. Although, as Black argues^30^ the goal of immunisation programmes is primarily to prevent severe disease and death.

Severe RSV infection early in life might be linked to later development of chronic conditions such as wheezing and asthma.^31^ Such long-term chronic conditions can be influential in cost-effectiveness analyses because of their long-term implications. However the relation between RSV infection and long-term outcomes is uncertain and has only been most clearly described for preterm infants.^32^ This additional complication was not included in our analysis but should be considered, particularly when more is known about the likely groups indicated for vaccination and the parameters of the relation between RSV and its sequelae.

RSV burden is substantial in children under 5 years, particularly in young infants. Passive or active immunisation directed at pregnant women, neonates, or infants could reduce this burden and would be good value for money if priced appropriately. There is potential for an RSV vaccine that protects infants and young children to be cost-effective because of the high disease burden in these groups. A maternal or newborn vaccination strategy is likely to avert the most severe disease and deaths, especially if it can be targeted at protecting infants born during the RSV season between late autumn and early spring. Vaccination of older children with a long-lasting vaccine might avert more health-care costs and episodes of mild disease. Our conclusions are based on ecological analyses of syndromic and laboratory data with economic modelling using a range of characteristics of potential prophylactic interventions. They will need to be validated when results from clinical trials and post-licensure studies become available.
