# Supplementary material for: Burden of paediatric respiratory syncytial virus disease and potential effect of different immunisation strategies: a modelling and cost-effectiveness analysis for England
Source: Lancet Public Health. 2017 Jul 31;2(8):e367–74. doi: 10.1016/S2468-2667(17)30103-2 (PMC5541134; doi:10.1016/S2468-2667(17)30103-2)
Supplement: Supplementary appendix [file mmc1.pdf]

THE LANCET
Public Health

Supplementary appendix

This appendix formed part of the original submission and has been peer reviewed. We post it as supplied by the authors.

Supplement to: Cromer D, van Hoek AJ, Newall AT, Pollard AJ, Jit M. Burden of paediatric respiratory syncytial virus disease and potential effect of different immunisation strategies: a modelling and cost-effectiveness analysis for England. *Lancet Public Health* 2017; **2:** e367–74.

The potential health and economic impact of future vaccines and prophylactic antibodies to prevent paediatric respiratory syncytial virus disease

Supplementary Materials

Disease Burden Estimation

Data sources

Most people who present to health care services with respiratory symptoms are not routinely tested for RSV. Hence primary care or hospital databases are not on their own a reliable estimate of RSV disease incidence. However, England collects data on both clinical attendances for acute respiratory infection, and separately, data on the organisms detected in microbiological testing for routine clinical purposes. Since the two databases are unlinked, we used statistical modeling to associate positive laboratory tests of numerous respiratory pathogens with general practice (GP) and hospital data on acute respiratory symptoms, based on the temporal trends in both datasets.

This approach has been used previously to estimate the burden of RSV and other seasonal infections(1-5). We adapted the method used to estimate influenza burden in England to inform the introduction of paediatric influenza vaccine in 2014; data and methods are described in more detail in our previous publication(1). For simplicity, we used data from the pre-2009 influenza pandemic era covering eight years (by week) from 2000/1 to 2007/8. Results of microbiological testing was obtained from Public Health England's LabBase2 database for children under five years of age and included positive tests for influenza A, influenza B, respiratory syncytial virus, parainfluenza, adenovirus, rhinovirus, *Streptococcus pneumoniae, Mycoplasma pneumoniae* and *Haemophilus influenzae*(6). Weekly GP consultations for acute respiratory disease consultations were obtained from the Royal College of General Practitioners Weekly Returns Service (RCGP), a national sentinel surveillance system, and scaled by the size of the population covered by the Royal College of General Practitioners practices to give weekly consultation rates per 100,000 people. We used all consultations reported in RCGP as either upper respiratory tract infection or lower respiratory tract infection. Corresponding weekly inpatient admissions to National Service Hospitals in England for acute respiratory disease were obtained from the Hospital Episode

Statistics (HES) database(7). Patients were included in the analysis if they had an acute respiratory illness code (ICD-10 codes J0*, J1*, J2*, J3*, J40*, J41*, J42*, J43*, J44*, J47*) in any diagnosis field, however they were not double counted if they had one of these codes in more than one field. The number of deaths in hospital by age and clinical risk group was estimated by counting inpatient admissions with an acute respiratory illness code extracted from the HES database with death recorded as the discharge method. Only deaths within 30 days of admission were included in the analysis.

Statistical Modeling

Weekly counts in laboratory reports for pathogens potentially responsible for acute respiratory illness were used as explanatory variables to estimate the proportion of health care outcomes (GP consultations, hospital admissions and deaths in hospital coded as acute respiratory illness) attributable to RSV. We used the same model, dataset and age-groups that were previously used in(1). Briefly, this was a generalized linear model for negative binomial outcome distributions with an identity link function. The model incorporated both a moving average to smooth fluctuations in laboratory reports and a linear term to account for secular trends in reporting and laboratory methods. The model equation is given in equation 1.

$$H_w^j = B_0^j + B_1^j w + \sum_p B_p^j r_{p,w}^j \qquad \text{Equation 1}$$

Here, H_w^j represents the number of reported outcomes (eg. hospitalisations) for age group *j* in week *w*. The right hand side is made up of constant term (B_0^j) representing outcomes attributed to causes other than the pathogens recorded in microbiological surveillance, a linear trend term that takes into account the change in outcomes over time (i.e. a coefficient, B_1^j, multiplied by the week number, *w*, which starts at 0 in the first week of the series), and the sum of the product of the number of laboratory reports reported in microbiological surveillance for each pathogen *p* in age group *j* in week *i* ($r_{p,w}^j$) and their coefficient, B_p^j. The coefficients are estimated by negative binomial regression using an identity link function. If the coefficients B_p^j are estimated to be negative, the term for the corresponding pathogen is removed for reasons of biological implausibility, and the model is re-run to obtain new estimates for the other coefficients. Backwards stepwise regression is used, meaning that the least significant pathogens is sequentially removed from the model provided it is deemed to have a significance level above 0.05 and the model is re-run to obtain new estimates for the other coefficients. The total non-pathogen related coefficient (sum of the baseline coefficient and the linear trend term, $B_0^j + B_1^j w$) is also required to be greater than zero for all values of *w* in the data series. We note that the inclusion of all respiratory pathogens in our regression

model differentiates this model from that of Taylor et al (5) who included only influenza and RSV. This means that we are able to accurately model the observed health care outcomes with only two non-pathogen related parameters, namely a constant term and a linear temporal term, and do not require any of the trigonometric or higher order polynomial terms included by Taylor et al.

Data were modeled in two groups – data from children under six months of age and data from children between six months and five years of age, resulting in the estimated weekly rate per 100,000 children of RSV attributable GP consultations, hospitalisations and deaths in hospital. Weekly numbers were then summed to give monthly RSV incidence rates by age group.

Results of Regression Burden Estimation

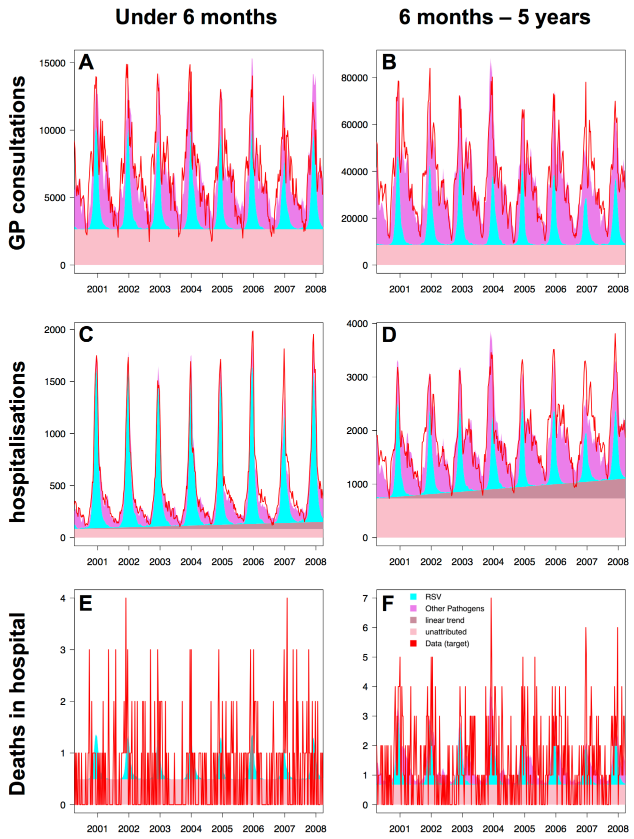

Supplementary Figure 1 Results of regression fitting to (A-B) GP consultations, (C-D) Hospitalisations and (E-F) Deaths in Hospital

(A,C and E) Show results of fitting to the under 6 month age group. (B,D and F) show results of fitting to the 6 month – 5 year age group.

Age stratification of RSV disease

To determine the age specific incidence of RSV attributable primary care outcomes, hospitalisations and deaths, we initially fit data in two age groups – those under six months of age, and those between six months and five years of age, as this was the age stratification most commonly used in public health data sources. However even within these age groups, studies suggest that RSV disease is concentrated in the very youngest children(8-12). As we did not have more finely stratified data on respiratory pathogens or outcomes in children as inputs for our regression analysis, we derived an estimate for the distribution of RSV hospitalisations by month of age based on existing studies. We fit a power function with an additional parameter for the first data point simultaneously to the proportion of hospitalisations by age group from 0 – 24 months of age using data from six studies in high income countries(8-14) . We used this function, shown in Supplementary Figure 2A and B to provide an estimate for the age distribution of cases. We then applied this function describing the distribution of cases by age to the estimated number of RSV hospitalisations obtained from the two broader age groups, to determine the actual number of cases for each month of age. To estimate the age distribution of GP consultations and deaths in hospital we used the same power function, however also applied a linear scaling factor to ensure that the proportion of cases in children under 6 months of age and between 6 months and 5 years matched our regression results. (Supplementary Figure 2). Uncertainty in the age distribution was not included in our modelling.

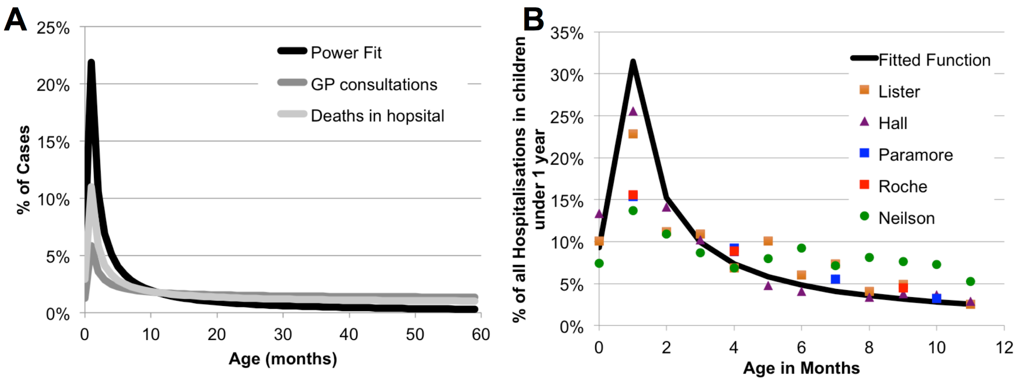

Supplementary Figure 2 (A) Fitted Age distribution of cases in children under 5 years of age. (B) Fitted results to proportion of hospitalisations in children under one year of age.

Description of the cost effectiveness model

Our static cohort model tracked monthly cohorts of children from birth to five years of age, differentiating them by their month of birth to capture the differences in RSV disease burden in children born at different times of the year. We then compared disease incidence in the presence of paediatric or maternal interventions, with the counterfactual of no such interventions, which was assumed to retain the current burden of disease.

The net cost of each intervention was calculated as the cost of vaccinating eligible individuals, minus the cost saving as a result of prevented disease. To allow comparability with other disease areas, we converted the health gains in children from each intervention into quality adjusted life years (QALYs) as a generic measure of health utility. We then estimated the potential cost-effectiveness of an intervention as the incremental cost of that intervention divided by the incremental QALYs gained by the intervention. For these calculations, we used the economic reference case prescribed by England's National Institute for Health and Care Excellence(15) for our economic methodology. In particular, we discounted costs and QALYs by 3.5% per year (and 1.5% in sensitivity analysis) and used a health care provider perspective.

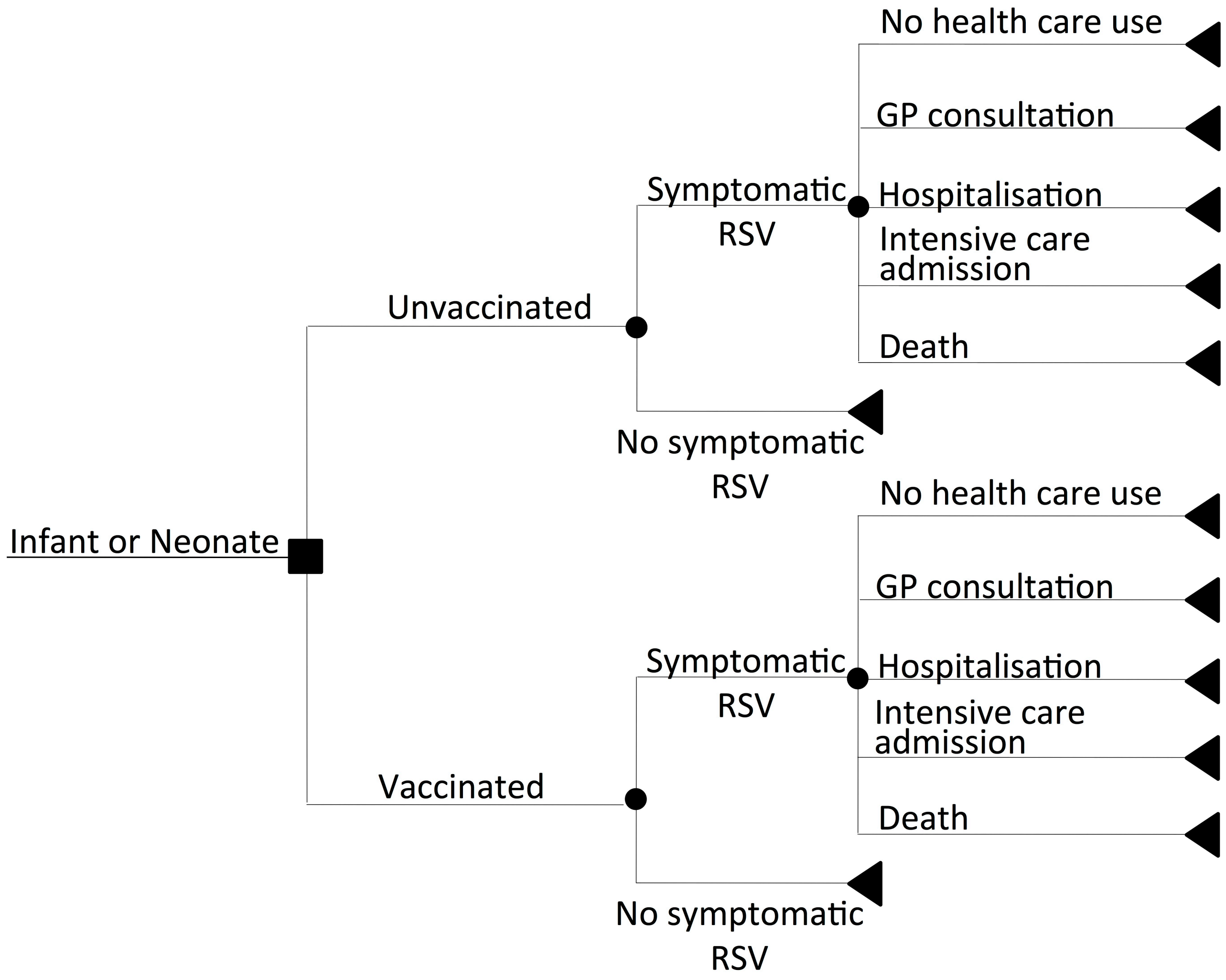

Supplementary Figure 3 Decision tree for immunisation against RSV

The probability of each branch depends on the immunisation strategy being considered, the month of birth of the child and the incidence of RSV at a given time of year. Note that it is possible for a patient to use more than one healthcare outcome, though this is not shown.

Cost and health-related quality of life parameters

The cost of treating RSV disease was estimated from English health care costs in 2014, including standard English reference costs for GP consultations(16) and hospitalisation admissions for Paediatric Acute Bronchiolitis (Reference-code PD15)(17). QALY loss for RSV disease leading to a GP consultation or hospitalisation was set on 0.01 and 0.04 respectively based on a previous cost-effectiveness analysis for the Netherlands(18). For patients admitted to intensive care, the QALY loss was set to 0.08(18). Life years lost were weighted according to QALYs from population norms(19) and discounted appropriately. A complete list of parameters used in the cost effectiveness model can be found in Table 1 of the manuscript

Life years lost

The risk of mortality from RSV is very low, and linked to severe co-morbidities. Thus, in addition to a scenario that incorporated RSV mortality, we also considered a scenario where mortality due to RSV was negligible. We calculated the number of life years lost as a result of RSV using the life expectancy of a child at the age of the RSV-associated death, based on data from England 2012-2014(20). These life years lost were converted into QALYs lost by multiplying each year of life lost with the average quality of life of English people at that age(19).

Intervention Strategies

Infant vaccination program ("infant strategy")

For this vaccination program, we assumed that vaccinated infants were protected from 3 months of age. To capture the most optimistic possibility, we assumed that for the infant strategy, once initiated vaccine induced immunity did not wane over the first five years of life.

Maternal / newborn strategies

An alternative strategy, is to protect infants from birth either though antibody transfer vira a maternal vaccine (maternal strategy) or a birth dose of a long-lasting antibody (newborn strategy). The disadvantage of this may be that protection via such interventions is unlikely to last as long. For these strategies, we assumed that a fixed proportion of infants receiving the intervention were protected from RSV disease from birth until its effect completely disappeared at six months of age. This is consistent with the duration of protection previously reported from naturally acquired maternal antibodies(21), with maternal and birth dose vaccines and antibodies in current development(22, 23) and with targeted protection reported from long-lived next-generation antibodies(24). We conservatively considered only protection to the infant and not the mother.

Combined Strategies

A combined strategy combines the infant strategy with either a maternal or newborn strategy. This may be considered to allow protection directly from birth and for a longer period within childhood. For this modification, we assumed that the level of protection afforded by the vaccine was the stronger of the protection induced by either strategy.

Switching on and off a newborn strategy

We assessed the cost effectiveness of a newborn strategy that was administered only to protect children born in certain months of the year. For this we considered 122 different

vaccination strategies that included 121 options in which women were vaccinated starting in each of 12 months of the year and ending between 1 and 11 months after that, as well as one strategy involving offering vaccination throughout the year.

Comparison with other studies

Our RSV-attributable burden of disease are similar to estimates provided in earlier studies.

RSV attributable hospitalisations

A study of RSV disease in Shropshire, England concluded that there were 3.6 hospital cases per 100 children under 6 months of age and 1.4 cases per child under one year of age(25). This is similar to our estimates of 4.4/100 and 2.9/100 for children under 6 months and one year respectively. Similarly, previous regression analyses by Taylor et. al.(5), Mueller et. al.(26) and Reeves et. al.(27) relating hospitalisations to laboratory reports estimated 4/100 children under 6 months(5), 2.8/100(26) or 3.5/100(27) in children under one and 0.4/100 children between 6 months and 5 years(5) (or 0.5/100 in children between 1 and 4 years(27)) which are almost identical to our estimates despite the use of either older(26) or newer(27) data or a restricted regression model(5). We observe that both our estimates and those of Taylor et al and Reeves et al for RSV attributable hospitalisations in children aged between 1 and 5 years of age are lower than Mueller et al. (0.38/100 children vs 1.3/100 children us vs Mueller et al). Our case fatality estimates are also lower than those reported by Mueller et al (0.94 vs 2.8 deaths per 1000 hospitalisations in children under 5 years of age)(26), though our case fatality estimates are in broad agreement with those of Hardelid et. al.(28) This may be due to the fact that treatment of RSV has improved in the 10 years between the study periods, or that the population is generally healthier. Our estimates of RSV attributable hospitalisations in children under 1, 2 and 5 years (2.9/100, 1.8/100 and 0.9/100 respectively) are in agreement with rates reported for severe RSV associated respiratory infections in Western Europe in a recent meta-analysis(29) and with rates reported in individual countries in Western Europe(30). We observe that they are significantly lower than rates reported in Spain, and thus a vaccine introduced in Spain may be even more cost effective than our estimates show.

RSV attributable GP consultations

Our estimates for RSV attributable GP consultations are also similar to estimates in a recent study of UK based data(5) (22/100 vs 14/100 RSV attributable GP visits in children under 6

months and 10.9/100 vs 9/100 in children between 6 months and 5 years, us vs the previous study).

Supplementary Tables

Parameter	Value	Source
Life expectancy at birth	81.62 years	(20)
Quality-adjusted life expectancy, undiscounted*	65.81	(19, 20)
Quality-adjusted life expectancy, discounted at 1.5% a year	38.91	(19, 20)
Quality-adjusted life expectancy, discounted at 3.5% a year	23.29	(19, 20)
Parameters applicable to preterm infants		
Proportion of cases occurring in pre-term infants	16.2%	(25)
Proportion of hospitalisations requiring intensive care admission or ventilation among non pre-term infants or infants over 1 year	1.76%	(25)
Proportion of hospitalisations requiring intensive care admission or ventilation among pre-term infants under 1 year	8.96%	(25)
Costs assumptions		
Cost of a GP visit	£46 (normal, SD= 1)	(16), SD chosen to be 2% of the mean, as for ICU admission.
Cost of a hospital admission	£1283 (normal, SD=£3.39)	(17)<br>Paediatric Acute Bronchiolitis (Reference-code PD15)
Extra cost of ventilation or intensive care admission	£6637.50 (normal, SD=£157.50)<br>(£1475 per day, normal SD=£35)	(17)<br>Paediatric advance critical care 5 with an average duration of 4.5 days.<br>(25)
QALYs lost per RSV disease episode		

Disease requiring GP consultation	0.01 (gamma, SD=.001)	(18)
Disease requiring hospital admission	0.04 (gamma, SD=.004)	(18)
Disease requiring intensive care admission or ventilation	0.08 (gamma, SD=.007)	(18, 31)

* Children under 10 were assumed to have an average QALY weight of 0.9

Supplementary Table 1 Parameters used in the cost effectiveness model

**Immunisation Strategy**	**Efficacy**					
	**50%**	**60%**	**70%**	**80%**	**90%**	**100%**
**Maternal**	£27	£32	£37	£43	£48	£54
**Newborn**	£40	£48	£56	£64	£72	£81
**Infant**	£96	£115	£135	£154	£173	£192
**Newborn+Infant**	£123	£148	£172	£197	£221	£246

Supplementary Table 2 Maximum cost effective price payable per fully vaccinated person for a range of different immunisation strategies and vaccine efficacies.

References

1. Cromer D, van Hoek AJ, Jit M, Edmunds WJ, Fleming D, Miller E. The burden of influenza in England by age and clinical risk group: A statistical analysis to inform vaccine policy. J Infect. 2014;68(4):363-71.
2. Harris J, Jit M, Cooper D, Edmunds W. Evaluating rotavirus vaccination in England and Wales. Part I. Estimating the burden of disease. Vaccine. 2007;25(20):3962-70.
3. Pitman R, Melegaro A, Gelb D, Siddiqui M, Gay N, Edmunds W. Assessing the burden of influenza and other respiratory infections in England and Wales. JInfect. 2007;54(6):530-8.
4. Zhou H, Thompson WW, Viboud CG, Ringholz CM, Cheng P-Y, Steiner C, et al. Hospitalizations associated with influenza and respiratory syncytial virus in the United States, 1993-2008. Clinical infectious diseases : an official publication of the Infectious Diseases Society of America. 2012;54(10):1427-36.
5. Taylor S, Taylor RJ, Lustig RL, Schuck-Paim C, Haguinet F, Webb DJ, et al. Modelling estimates of the burden of respiratory syncytial virus infection in children in the UK. BMJ open. 2016;6(6):e009337.
6. Grant AD, Eke B. Application of information technology to the laboratory reporting of communicable disease in England and Wales. Communicable disease report CDR review. 1993;3(6):R75-8.
7. The Health and Social Care Information Centre. HES Online 2013 [updated Jul 18. Available from: http://www.hscic.gov.uk/hes.
8. Ranmuthugala G, Brown L, Lidbury BA. Respiratory syncytial virus--the unrecognised cause of health and economic burden among young children in Australia. Communicable diseases intelligence quarterly report. 2011;35(2):177-84.
9. Roche P, Halliday L, O'Brien E, Spencer J. The Laboratory Virology and Serology Reporting Scheme, 1991 to 2000. Communicable diseases intelligence quarterly report. 2002;26(3):323-74.

10. Paramore LC, Ciuryla V, Ciesla G, Liu L. Economic impact of respiratory syncytial virus-related illness in the US: an analysis of national databases. PharmacoEconomics. 2004;22(5):275-84.
11. Lister S, McIntyre P, Menzies R. The epidemiology of respiratory syncytial virus infections in New South Wales children, 1992-1997. New South Wales public health bulletin. 2000;11(7):119-23.
12. Hall CB, Weinberg GA, Blumkin AK, Edwards KM, Staat MA, Schultz AF, et al. Respiratory syncytial virus-associated hospitalizations among children less than 24 months of age. Pediatrics. 2013;132(2):e341-8.
13. Nielsen HE, Siersma V, Andersen S, Gahrn-Hansen B, Mordhorst CH, Nørgaard-Pedersen B, et al. Respiratory syncytial virus infection--risk factors for hospital admission: a case-control study. Acta paediatrica (Oslo, Norway : 1992). 2003;92(11):1314-21.
14. Rietveld E, De Jonge HCC, Polder JJ, Vergouwe Y, Veeze HJ, Moll HA, et al. Anticipated costs of hospitalization for respiratory syncytial virus infection in young children at risk. The Pediatric infectious disease journal. 2004;23(6):523-9.
15. NICE. Guide to the methods of technology appraisal 2013. 2013.
16. Curtis L. Unit Costs of Health and Social Care 2014. 2014.
17. Health UDo. NHS reference costs 2013 to 2014. 2014.
18. Meijboom M, Rozenbaum MH, Benedictus A, Luytjes W, Kneyber MCJ, Wilschut JC, et al. Cost-effectiveness of potential infant vaccination against respiratory syncytial virus infection in The Netherlands. Vaccine. 2012;30(31):4691-700.
19. van den Berg B. Sf-6d population norms. Health economics. 2012;21(12):1508-12.
20. ONS. National Life Tables, England & Wales, 1980-82 to 2012-14. 2014.
21. Ochola R, Sande C, Fegan G, Scott PD, Medley GF, Cane PA, et al. The level and duration of RSV-specific maternal IgG in infants in Kilifi Kenya. PloS one. 2009;4(12):e8088.
22. Dubovsky F. Medimmune’s RSV Product Development Overview. WHO Consultation on respiratory syncytial virus (RSV) vaccine development2015.
23. Mazur NI, Martinón-Torres F, Baraldi E, Fauroux B, Greenough A, Heikkinen T, et al. Lower respiratory tract infection caused by respiratory syncytial virus: current management and new therapeutics. The Lancet Respiratory medicine. 2015;3(11):888-900.
24. Higgins D, Trujillo C, Keech C. Advances in RSV vaccine research and development - A global agenda. Vaccine. 2016.
25. Deshpande SA, Northern V. The clinical and health economic burden of respiratory syncytial virus disease among children under 2 years of age in a defined geographical area. Archives of disease in childhood. 2003;88(12):1065-9.
26. Müller-Pebody B, Edmunds WJ, Zambon MC, Gay NJ, Crowcroft NS. Contribution of RSV to bronchiolitis and pneumonia-associated hospitalizations in English children, April 1995-March 1998. Epidemiology and infection. 2002;129(1):99-106.
27. Reeves RM, Hardelid P, Gilbert R, Warburton F, Ellis J, Pebody RG. Estimating the burden of respiratory syncytial virus (RSV) on respiratory hospital admissions in children less than five years of age in England, 2007-2012. Influenza and other respiratory viruses. 2017;11(2):122-9.
28. Hardelid P, Pebody R, Andrews N. Mortality caused by influenza and respiratory syncytial virus by age group in England and Wales 1999-2010. Influenza and other respiratory viruses. 2013;7(1):35-45.
29. Nair H, Nokes DJ, Gessner BD, Dherani M, Madhi SA, Singleton RJ, et al. Global burden of acute lower respiratory infections due to respiratory syncytial virus in young children: a systematic review and meta-analysis. Lancet (London, England). 2010;375(9725):1545-55.
30. Bont L, Checchia PA, Fauroux B, Figueras-Aloy J, Manzoni P, Paes B, et al. Defining the Epidemiology and Burden of Severe Respiratory Syncytial Virus Infection Among Infants and Children in Western Countries. Infectious diseases and therapy. 2016;5(3):271-98.
31. Greenough A, Alexander J, Burgess S, Bytham J, Chetcuti PAJ, Hagan J, et al. Health care utilisation of prematurely born, preschool children related to hospitalisation for RSV infection. Archives of disease in childhood. 2004;89(7):673-8.
